# Case of Abscess at the Neck of the Bladder, Causing Retention of the Urine

**Published:** 1844-10

**Authors:** Wm. B. Herrick

**Affiliations:** Lecturer on Anatomy in the Rush Medical College


*Case of Abscess at the Neck of the Bladder, causing retention of the Urine.* By Wm. B. Herrick, M. D., Lecturer on Anatomy in the Rush Medical College.

In December of last year, I was called to A. M., a man forty-five years of age, who had been suffering for eight days previous to my visit with intense pain, heat and a sense of fullness in the region above the perineum, accompanied with gradually increasing painful and difficult micturition and defecation. This condition of things ended in retention of urine, which had continued during the twenty-four hours previous to my arrival. It also appeared from a further history of the case, that the above named symptoms came on soon after the patient's return from a long journey on horseback, and that two quacks were first called, who gave lobelia emetics, cayenne, and an immense quantity of what they called "gravel root tea," at short intervals for six days and nights; thus exhausting the patient, aggravating inflammatory action, and increasing the secretion of urine to such an extent as to distend the bladder to its utmost dimensions.

I found the man exhausted, surface bedewed with cold perspiration, countenance sunken, and with a weak pulse at 120.

Upon examination, the bladder was felt above the pubis, tender and greatly distended; while a finger passed up the rectum discovered a globular tumor from two to three inches in diameter, distinctly fluctuating at every point that could be reached by the finger, occupying the situation of the prostate gland at the neck of the bladder; thus pressing that viscus upwards, elongating the urethra, and crowding its membranous portion forward against the arch of the pubis.

As nothing could be felt like a lobulated structure, and as the tumor seemed to extend itself equally in all directions from the natural situation of the prostate, I concluded that this was a case of abcess formed in the cellular structure within and around that organ. There is evidence of the correctness of this opinion in the following observations of Mr. Cooper, who says: "When an abscess follows inflammation of the prostate, the body of the gland itself does not suppurate, but only the surrounding parts and the cellular substance which connects its lobes together.—This, at least, was what was observed in examining several dead subjects who were publicly opened in the amphitheatre of the Hotel Dieu."

In the above case, the most distressing and dangerous symptoms were those caused by retention of urine; but it was found impossible to pass a catheter further than through the membranous portion of the urethra, where it came in contact with the tumor, which, when pressed on, could be felt fluctuating beneath the extremity of the instrument; hence it appeared necessary first to open the abscess. "Should the abscess lie near the rectum and perineum, and admit of being distinctly felt, Desault conceived that a free opening would expedite the cure." "Several cases of this description," have been "treated in this way with success."* In accordance with the above recommendation, a free opening into the rectum might have been made in this case, but such a course seemed to me objectionable, on account of the danger of extravasation of pus into the loose cellular substance between the rectum and bladder. I therefore determined to avoid this difficulty by opening the abscess, if possible, into the excretory canal to which the gland is most firmly attached. In accordance with this determination, a silver catheter, with a point not very blunt, was selected, and passed up the urethra till it came in contact with the tumor; then, by making moderate pressure, its beak was forced from the urethra into its cavity, thus giving exit to a large quantity of pus through the instrument. It was then partially withdrawn, and with the hand slightly depressed, pushed gently onward through the natural passage into the bladder, and an immense quantity of urine drawn off. The pain, and other unfavorable symptoms were almost immediately relieved, and, after passing the catheter once or twice more, first into the abscess, then into the bladder to relieve them of their contents, the cavity of the abscess contracted rapidly, and all obstructions to the free passage of urine, together with the attendant difficulties, soon disappeared.

The above case may be interesting to the profession, as showing the importance of ascertaining the true cause of the difficulty in all cases of retention of urine; and from the fact that abscesses within and around the prostate are not of frequent occurrence. It also shows that simple abscess of this structure, arising from acute inflammation, when not complicated with structure of the urethra or other diseases, is not, if treated promptly, attended with much danger.

---

### PRACTICAL MEDICINE, &c.

*Scarification of the Gums during Dentition.* By Marshall Hall, M. D., F. R. S., &c.—There is no practical fact of the truth and value of which I am more satisfied than that of the effect and efficacy of scarification of the gums in infants, and not in infants only, but in children. But the prevailing, I may say the univer-

*Cooper's Surg'l Dict.; Art. Prostate Gland.

